# The SIRT1-c-Myc axis in regulation of stem cells

**DOI:** 10.3389/fcell.2023.1236968

**Published:** 2023-07-24

**Authors:** Wei Fan, Xiaoling Li

**Affiliations:** Signal Transduction Laboratory, National Institute of Environmental Health Sciences, Research Triangle Park, Durham, NC, United States

**Keywords:** c-Myc, SIRT1, stem cells, deacetylation, pluripotency, differentiation, c-Myc/Max heterodimer, positive feedback loop

## Abstract

SIRT1 is the most conserved mammalian NAD^+^-dependent protein deacetylase. Through deacetylation of transcriptional factors and co-factors, this protein modification enzyme is critically involved in metabolic and epigenetic regulation of stem cells, which is functionally important in maintaining their pluripotency and regulating their differentiation. C-Myc, a key member of Myc proton-oncogene family, is a pivotal factor for transcriptional regulation of genes that control acquisition and maintenance of stemness. Previous cancer research has revealed an intriguing positive feedback loop between SIRT1 and c-Myc that is crucial in tumorigenesis. Recent literature has uncovered important functions of this axis in regulation of maintenance and differentiation of stem cells, including pluripotent stem cells and cancer stem cells. This review highlights recent advances of the SIRT1-c-Myc axis in stem cells.

## 1 Introduction

Stem cells, including pluripotent stem cells (PSCs), adult stem cells (ASCs), and cancer stem cells (CSCs), possess the ability to self-renew and to differentiate to give rise to all cell types in organs, tissues, or tumors. Embryonic stem cells (ESCs) and induced pluripotent stem cell (iPSCs) are two types of PSCs. ESCs are derived from the inner cell mass of a blastocyst (early stage of preimplantation embryos). iPSCs can be induced *in vitro* from adult somatic cells, such as murine embryonic fibroblasts (MEFs) or human somatic cells, through simultaneous overexpression of core pluripotent factors including OCT4, SOX2, KLF4, and c-Myc (Takahashi and Yamanaka, 2006; Smith and Dalton, 2010). These cells can be unlimitedly expanded *in vitro* while maintaining their pluripotency indefinitely.

C-Myc is one of the key pluripotent factors. C-Myc was firstly discovered as an oncogene that belongs to the Myc family of proton-oncoproteins. This family of proton-oncoproteins contains three main transcription factors, c-Myc, N-Myc, and L-Myc. They are basic-helix-loop-helix/leucine zipper (bHLH) DNA binding proteins and are known to be fundamentally important for a number of cellular activities, such as metabolism, apoptosis, proliferation and differentiation (Prendergast, 1999; Meyer and Penn, 2008; Dang, 2013; Bretones et al., 2015). In healthy cells, maintaining an appropriate abundance and activity of MYC proteins is critical for these cellular programs. Aberrations or upregulation of MYC-related pathways by alternate mechanisms are observed in the vast majority of cancers (Dhanasekaran et al., 2021). Specifically, dysregulations of MYC proteins are associated with 70% of human cancers, and a wealth of evidence suggests that aberrantly expressed MYC proteins are closely related with both tumor initiation and maintenance (Llombart and Mansour, 2022). As the first member discovered in Myc family, c-Myc contributes to the genesis of many human cancers and is associated with alteration of cellular metabolism (Dang et al., 2009). Mechanistically, c-Myc controls global gene expression, especially genes involved in the biogenesis of ribosomes and mitochondria. These actions in turn impact cell proliferation, differentiation, cell cycle, apoptosis, as wells as metabolism of glucose and glutamine in cancer cells (Dang et al., 2009). In PSCs, c-Myc also acts as a transcriptional factor to regulate several thousand genes involved in cell reprogramming as well as maintenance and establishment of the pluripotent state (Chappell and Dalton, 2013). Additionally, c-Myc is important in embryogenesis. Its expression is maintained at the highest level during embryonic stage, declines over development, and eventually stays relatively low in mature organs (Elbadawy et al., 2019).

The activation of c-Myc is modulated by post-translational modifications, such as phosphorylation, de/acetylation and ubiquitination (Gregory and Hann, 2000; Faiola et al., 2005). For instance, c-Myc is acetylated by HATs (histone acetyltransferase) and its acetylation status has a complex impact on its protein stability and subsequent transcriptional activity (Faiola et al., 2005). SIRT1, a highly conserved nicotinamide adenosine dinucleotide (NAD^+^) dependent class III histone deacetylase, is able to interact with and deacetylate c-Myc in cancer cells, which in turn increase its stability and activity (Mao et al., 2011; Menssen et al., 2012).

SIRT1 is the most conserved mammalian member of the Silent Information Regulator 2 (Sir2) family known as sirtuins (Calvanese et al., 2010; Vassilopoulos et al., 2011). The deacetylation activity of sirtuins is strictly dependent on NAD^+^, a cofactor for hundreds of metabolic reactions in all cell types. Sirtuins deacetylate target proteins by transferring a wide range of lipid acyl-groups, such as acetyl, succinyl, malonyl, glutaryl, or long-chain acyl-groups, from their protein substrates to the ADP-ribose moiety of NAD^+^(He et al., 2012; Choudhary et al., 2014; Wagner and Hirschey, 2014; Imai and Guarente, 2016). This exclusive NAD^+^ requirement makes SIRT1 an important cellular metabolic sensor and regulator. It can sense the alteration of cellular energy status to modulate the functions of a wide range of protein substrates, including transcription factors and co-factors, histones, metabolic enzymes, and cell membrane proteins (Fang et al., 2019).

SIRT1 is highly expressed in both mouse ESCs (mESCs) and human ESCs (hESCs) (Calvanese et al., 2010; Vassilopoulos et al., 2011; Tang et al., 2017). Recent studies have shown that through deacetylation of transcription factors and co-factors, particularly c-Myc, SIRT1 plays important roles in normal embryogenesis and mouse embryonic stem cell pluripotency maintenance (Tang et al., 2017; Fan et al., 2021). Intriguingly, activation of c-Myc can enhance expression, stability, and activation of SIRT1. SIRT1 and c-Myc thereby form a positive feedback loop for regulation of tumorigenesis (Menssen et al., 2012). This review article summarizes the latest knowledges on the SIRT1-c-Myc axis in regulation of acquisition and maintenance of stemness, the capability of self-renewal potential and multi-lineage differentiation, differentiation of stem cells, and embryogenesis.

## 2 C-Myc is critical for the self-renewal and pluripotency of ESCs and normal embryogenesis

C-Myc is a critical regulator of normal embryogenesis in mice. Early studies showed that mouse embryos derived from the homozygous *c-myc* mutant mESCs display the embryonic lethality between 9.5 and 10.5 days of gestation. The homozygous *N-myc* mutant mESCs derived mouse embryos are also embryonic lethal at around 11.5 days of gestation. Both *c-myc* and *N-myc* mutant embryos have severe multi-organ development defects (Yoshida, 2018). In mESCs, although neither *c-myc* nor *N-myc* is required for their maintenance and functions, mESCs with *c-myc* and *N-myc* genes simultaneously knocked out exhibit severe disruption in their self-renewal and pluripotency. These cells have reduced survival, along with enhanced differentiation (Varlakhanova et al., 2010). Consistently, chimeric embryos generated by injection of *c-myc* and *N-myc* doubly KO mESCs most often completely fail to develop or, in rare cases, survive but with severe defects (Varlakhanova et al., 2010). Therefore, *c-myc* and *N-myc* together are important in maintaining the pluripotency of mESCs by suppressing early stage differentiation (Yoshida, 2018).

**FIGURE 1:**
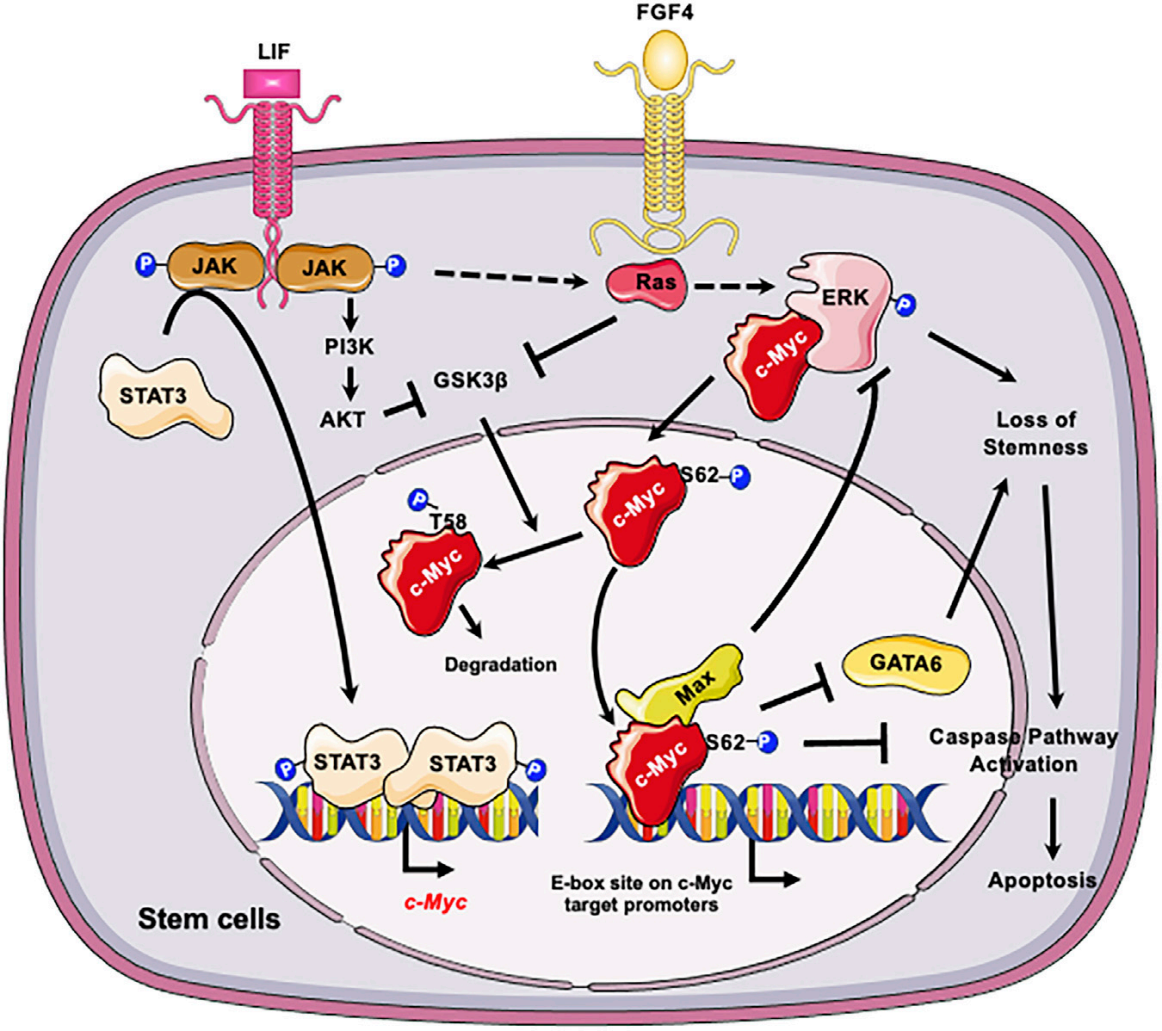
C-Myc is critical for the self-renewal and pluripotency of ESCs. LIF promotes the transcription of *c-myc* through JAK-STAT3 pathway and prevents GSK3β-mediated phosphorylation of c-Myc T58 and subsequent degradation. In parallel LIF and FGF4 activate the ERK1/2 signaling cascade, resulting in phosphorylation of c-Myc at S62. The phosphorylation enhances the stability of c-Myc, thereby promoting its interaction with Max and the formation of c-Myc/Max heterodimer complex. The c-Myc/Max complex in turn prevents loss of stemness of mESCs by feedback inhibition of p-ERK and suppression of GATA6 and suppresses subsequent apoptosis. Figures were created using images downloaded and adapted from Service Medical ART: SMART (https://smart.servier.com/image-set-download/). Servier Medical Art by Servier is licensed under a Creative Commons Attribution 3.0 Unported License (https://creativecommons.org/licenses/by/3.0/).”

At the molecular level, c-Myc is important for maintaining self-renewal and pluripotency of mESCs by interacting with leukemia inhibitory factor (LIF)/Signal transducer and activator of transcription 3 (STAT3) signal pathway (Cartwright et al., 2005). Specifically, LIF actives c-Myc via two mechanisms (Figure 1): elevates the transcription of *c-myc* through the Janus kinase (JAK)-STAT3 pathway and prevents GSK3β-mediated phosphorylation of c-Myc T58 and subsequent degradation (Cartwright et al., 2005). Moreover, the stability of c-Myc is sensitive to growth factors such as fibroblast growth factor 4 (FGF-4), which activates extracellular signal-regulated kinase (ERK1/2), a mitogen-activated protein kinase (MAPK). ERK phosphorylates c-Myc at Ser 62, leading to its stabilization (Sears et al., 2000; Lee et al., 2008; Ying et al., 2008). The phosphorylated c-Myc then interacts with Myc-associated protein X (Max) to form a heterodimer complex. This complex then binds to the “E-box” sequence in the target gene promoter region, thereby activating or repressing the transcription of target genes (Yoshida, 2018). Importantly, the c-Myc/Max heterodimer complex acts as a central node of the regulatory network which prevents loss of stemness of mESCs and subsequent apoptosis (Figure 1). Firstly, this complex inhibits the p-ERK, which forms a negative feedback loop to prevent the MARK signaling induced loss of stemness (Hishida et al., 2011). Secondly the c-Myc/Max complex can directly suppress expression of primitive endoderm master regulator, GATA6, to maintain stemness (Smith et al., 2010). Consistently, depletion of *Max* gene in mESCs results in loss of the undifferentiated state, upregulation of linage markers, and induction of apoptosis/death with Caspase-3 activation (Hishida et al., 2011). All these are primarily caused by activation of MAPK signaling, because inhibiting MAPK kinase signaling significantly blocks the decline of pluripotency genes and eliminates differentiated cells (Hishida et al., 2011).

## 3 SIRT1 regulates stem cell maintenance and embryogenesis at multiple levels

SIRT1 is highly expressed in the pre-implantation embryos and ESCs compared with adult tissues/cells (Tang et al., 2017). It plays an important role in maintaining normal embryogenesis and animal development. Mice with germline deletion of *Sirt1* display severe development defects, such as neonatal lethality, defective germ cell differentiation, developmental defects of the retina and heart, bone developmental delay, and intrauterine growth retardation (Cheng et al., 2003; McBurney et al., 2003; Wang et al., 2008; Tang et al., 2014; Liu et al., 2017).

Accumulating evidences indicate that SIRT1 regulates embryogenesis, animal development, and ESC pluripotency maintenance through multilevel mechanisms, which strictly rely on its protein deacetylation activity (Fang et al., 2019). The deacetylation substrates of SIRT1 in stem cells include a key component of core pluripotency network OCT4, tumor repressor p53, histones, and epigenetic regulator DNA methyltransferase 3-like (DNMT3L). For instance, it has been shown that SIRT1-mediated deacetylation of OCT4 is required to maintain the naïve state of mESCs, whereas SIRT1 reduction-induced acetylation of OCT4 leads to naïve-to-primed transition (Zhang et al., 2014; Williams et al., 2016). SIRT1 also modulates DNA methylation in stem cells through antagonizing *Dnmt3l* transcription and protein stability by deacetylation of histones and DNMT3L itself (transcriptionally and post-transcriptionally) (Heo et al., 2017). These actions of SIRT1 control the expression of imprinted and germline genes and the differentiation potential of mESCs, which are important in maintaining the normal neurogenesis and spermatogenesis (Heo et al., 2017). Moreover, SIRT1 represses the transcription of differentiation genes in ESCs through direct deacetylation of histones. Consequently, the reduction of SIRT1 reactivates those development genes during embryo developments (Calvanese et al., 2010). Furthermore, SIRT1 suppresses retinoic acid receptor (RAR)-mediated activation of differentiation genes in mESCs by deacetylation of a cellular retinoic acid binding protein II (CRABPII). Deacetylation recycles CRABPII from the nucleus out to the cytosol, thereby terminating the retinoic acid signaling (Tang et al., 2014). Additionally, SIRT1 is important to maintain healthy pluripotent ESCs. In response to endogenous reactive oxygen species (ROS), SIRT1 deacetylates p53 and promotes its mitochondrial translocation from the nucleus. This action of SIRT1 sensitizes mESCs to mitochondrial p53-induced apoptosis while inhibiting nuclear p53-mediated suppression of *Nanog* expression (Han et al., 2008). Together, by deacetylation of key regulators, SIRT1 acts as a pivotal regulator to orchestrate metabolic and epigenetic signal pathways to maintain pluripotent ESCs and normal embryogenesis.

## 4 The SIRT1-c-Myc feedback loop in regulation of tumorigenesis in cancer cells

The link between SIRT1 and c-Myc was first observed in cancer cells. It has been previously shown that both c-Myc and SIRT1 are highly elevated in major types of cancer cells, where c-Myc may elicit apoptosis or premature senescence through p53-dependent pathway (Vafa et al., 2002; Dominguez-Sola et al., 2007; Menssen et al., 2007; Campaner et al., 2010). Since SIRT1 is known to inhibit p53 through deacetylation (Luo et al., 2001; Vaziri et al., 2001; Langley et al., 2002), SIRT1 may regulate c-Myc activation through p53. Subsequent studies revealed that SIRT1 could directly activate the transactivation activity of c-Myc. To activate the transcription of its target genes, c-Myc needs to form a heterodimer with Max to recognize the E-box sequence in the target promoters (Yoshida, 2018; Singh et al., 2022). Mao et al. (2011) showed that SIRT1 binds to and deacetylates the C-terminal bHLH-ZIP motif containing region of c-Myc, which is directly involved in the formation of c-Myc/Max heterodimer. Deacetylation of c-Myc by SIRT1 increases its binding affinity to Max, presumably due to deacetylation induced conformation changes. The enhanced c-Myc/Max dimerization consequently facilitates the transcription of c-Myc target genes, such as human telomerase reverse transcriptase (*hTERT*), cyclinD2 (*CCND2*) and Lactate Dehydrogenase A (*LDHA*), thereby promoting cell proliferation (Mao et al., 2011). Deacetylation of c-Myc by SIRT1 also affects its stability in immortalized or cancer cells. Previous reports have shown that acetylation of c-Myc by PCAF and TIP60 inhibits its ubiquitination and subsequently increases its stability (Vervoorts et al., 2003; Patel et al., 2004). Consistently, Yuan et al. (2009) reported that SIRT1 deacetylates c-Myc at K323 and decreases its stability in immortalized cells. However, Menssen et al. (2012) reported that deacetylation of c-Myc by SIRT1 increases its stability and enhances its transcriptional activity. C-Myc can be conjugated with both lysine-48 (K48)- and lysine-63 (K63)-linked polyubiquitin chains, and K63-linked ubiquitination of c-Myc does not lead to its degradation. Instead, it is required for recruitment of the coactivator p300, transactivation of multiple target genes, and induction of cell proliferation by c-Myc (Adhikary et al., 2005). Menssen et al. (2012) showed that SIRT1-mediated deacetylation increases the conjugation of K63-linked ubiquitin chains to c-Myc, which in turn stabilizes c-Myc by competing with K48-likned degradative ubiquitination. The reasons for the discrepancies between studies of Yuan et al. (2009) and Menssen et al. (2012) are still not completely clear.

Conversely, c-Myc has also been reported to enhance the activity of SIRT1 through several different mechanisms. Firstly, c-Myc increases the NAD^+^/NADH ratio by transcriptional activation of nicotinamide-phosphoribosyltransferase (NAMPT), the rate-limiting enzyme of the amidated NAD^+^ salvage pathway (Menssen et al., 2012). Menssen et al. (2012) showed that the NAMPT promoter contains “E-box” binding motifs of c-Myc in the vicinity of the transcription start site (TSS). Activation of c-Myc transcriptionally increases the mRNA levels of NAMPT, which elevates cellular NAD^+^ salvage and subsequently promotes the activity of SIRT1. Secondly, c-Myc can enhance the activity of SIRT1 by sequestering its inhibitor deleted in breast cancer 1 (DBC1) (Menssen et al., 2012). DBC1 binds to the active site of SIRT1 and inhibits SIRT1–substrate interaction (Kim et al., 2008; Zhao et al., 2008). c-Myc also interacts with DBC1, which protects SIRT1 from interaction with DBC1, resulting in reduced inhibition of SIRT1 (Koch et al., 2007; Menssen et al., 2012). Finally, c-Myc can directly binds to the conserved “E-box” DNA binding motif on the *Sirt1* promoter and induces its transcription (Yuan et al., 2009). Interestingly, this transcriptional activation can be inhibited by p53, as p53 shares the response element with c-Myc and blocks the c-Myc recruitment on the *Sirt1* promoter (Yuan et al., 2017).

**FIGURE 2:**
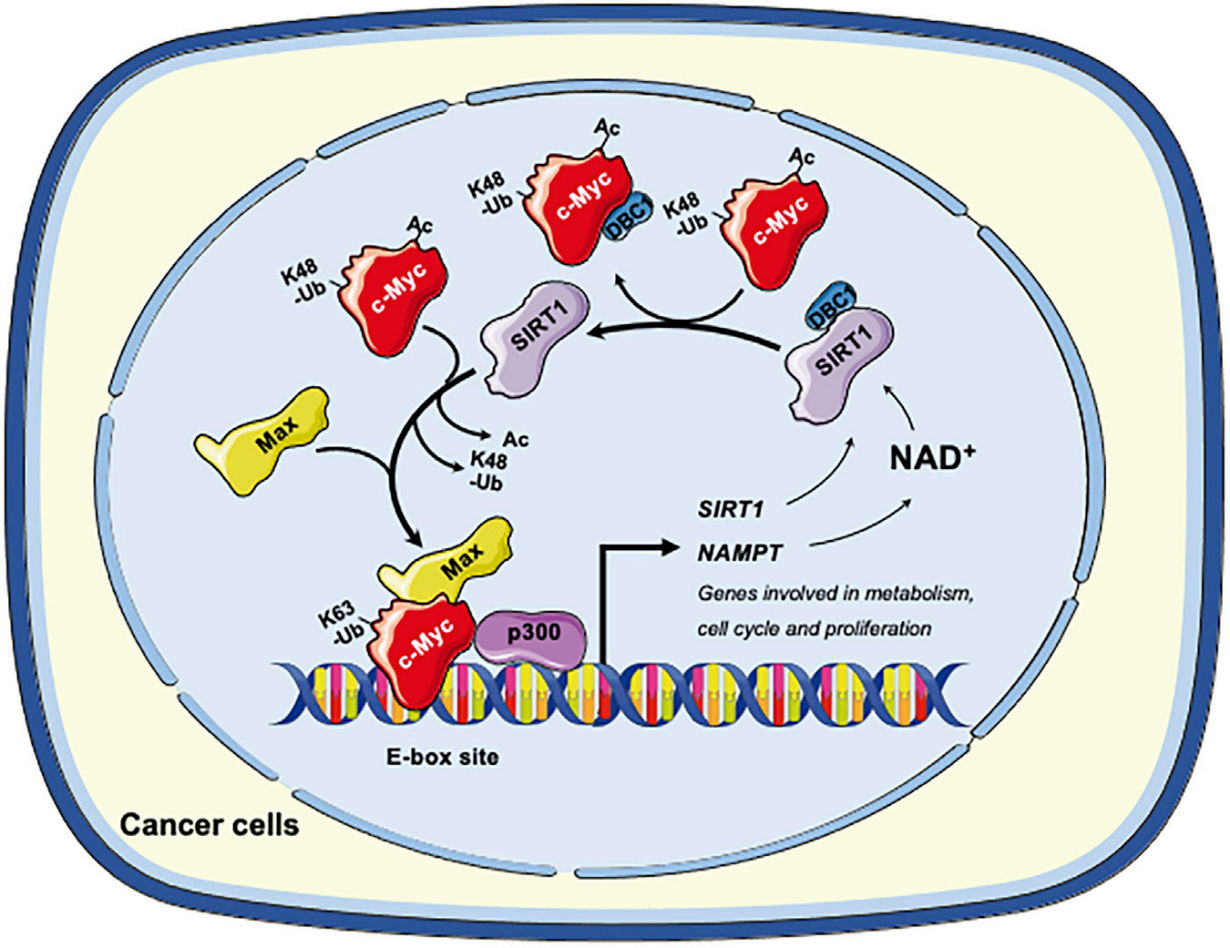
The SIRT1-c-Myc positive feedback loop in cancer cells. SIRT1-mediated deacetylation of c-Myc increases its binding affinity to Max. Deacetylation of c-Myc by SIRT1 also increases K63-linked polyubiquitination while repelling degradative K48-linked polyubiquitination, enhancing the stability of c-Myc and increasing recruitment of p300. Both mechanisms facilitate the transactivation of c-Myc target genes, including genes involved in metabolism, cell cycle and cell proliferation. Conversely, c-Myc also enhances the activity of SIRT1. Firstly, c-Myc increases the cellular NAD^+^ by transcriptional activation of NAMPT, the rate-limiting enzyme in the amidated NAD^+^ salvage pathway. Increased NAD^+^ enhances the deacetylase activity of SIRT1. Secondly, c-Myc sequesters the SIRT1 inhibitor DBC1, thereby increasing SIRT1-substrate interaction. Finally, c-Myc directly increases the transcription of SIRT in p53 deficient cells. Figures were created using images downloaded and adapted from Service Medical ART: SMART (https://smart.servier.com/image-set-download/). Servier Medical Art by Servier is licensed under a Creative Commons Attribution 3.0 Unported License (https://creativecommons.org/licenses/by/3.0/).”

Collectively, in cancer cells, SIRT1 and c-Myc could form a positive feedback loop, in which activation of c-Myc increases the expression and activity of SIRT1 to deacetylate c-Myc. Deacetylation of c-Myc increases its stability and transactivation activity (Figure 2). This axis of SIRT1-c-Myc positive feedback may orchestrate cellular response to endogenous or exogenous stimulations.

## 5 The SIRT-c-Myc axis is important in metabolic and epigenetic regulation of mESCs and mouse embryonic development

Given the importance of SIRT1 and c-Myc in regulation of stem cell self-renewal, pluripotency, and differentiation, it is not surprising that the SIRT1-c-Myc axis revealed in cancer research is also functionally important in stem cell biology and animal embryonic development.

The stemness of PSCs, including ESCs, is sustained by their specific metabolic programs and epigenetic status (Folmes et al., 2012; Zhang et al., 2012; Ito and Suda, 2014; Teslaa and Teitell, 2015). These special metabolic programs, including high glycolytic flux under aerobic condition, consumption of high levels of exogenous glutamine, as well as high dependence on one-carbon catabolism, are required to produce precursors and ATP for the high proliferation of PSCs. Moreover, the intermediate products of these metabolic processes, such as acetyl-CoA, NAD^+^, α-ketoglutarate, and S-adenosylmethionine (SAM), can also act as cofactor or co-substrates of enzymes which participate epigenic regulation of chromatin and gene expression in PSCs (Takahashi and Yamanaka, 2006; Wellen et al., 2009; Cai et al., 2011; Xu et al., 2011; Shyh-Chang et al., 2013; Moussaieff et al., 2015). Consequently, the distinctive metabolic programs in PSCs are directly linked to their unique epigenetics and gene expression profiles, thereby strongly influencing the self-renewal and pluripotency of PSCs (Folmes et al., 2011; Carey et al., 2015).

**FIGURE 3:**
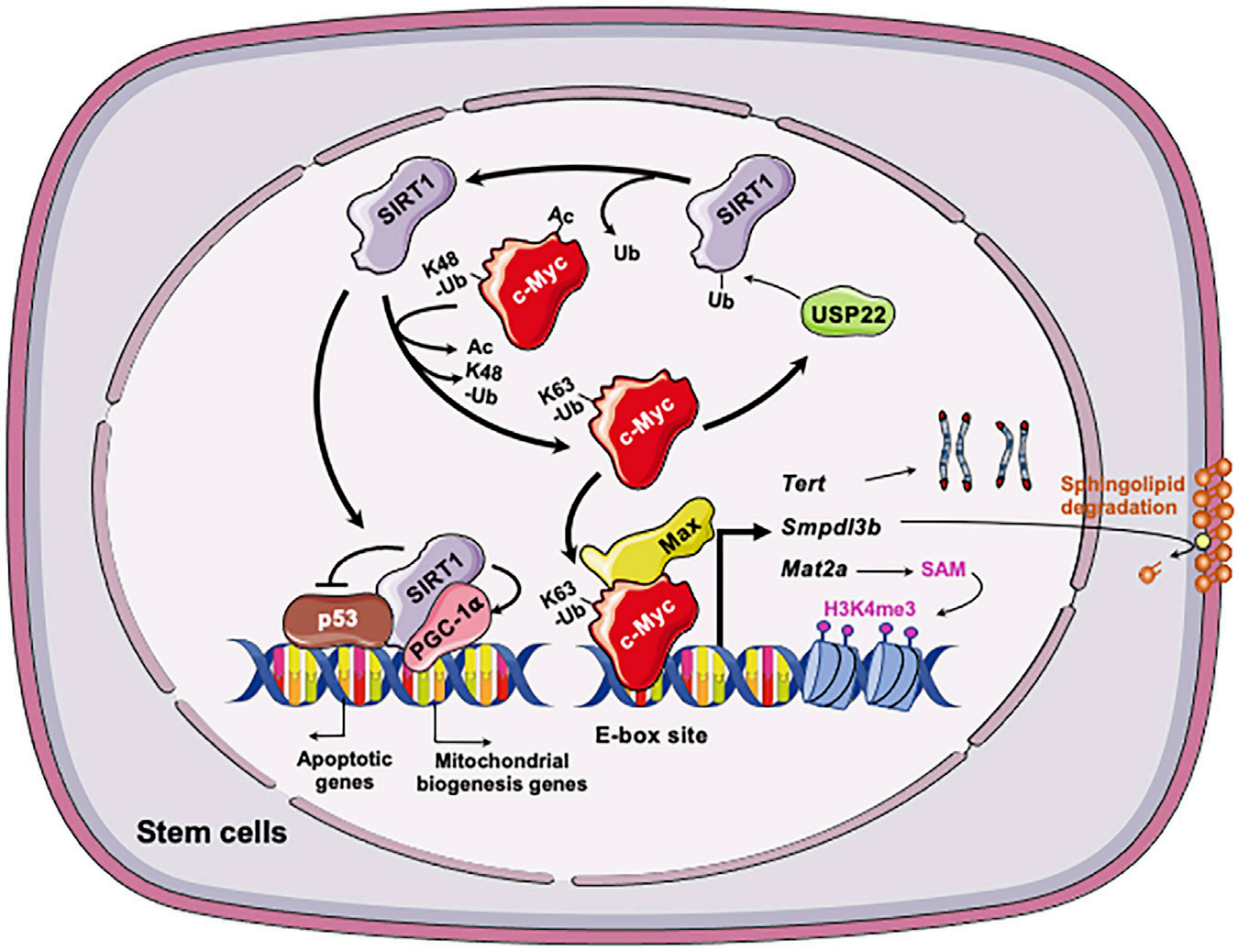
The SIRT1-c-Myc axis in regulation of stem cells. Both SIRT1 and c-Myc are highly expressed in stem cells, including mESCs, iPSCs, and LSCs. In all three types of stem cells, SIRT1 deacetylates c-Myc, which increases stability, presumably via reported exchange of K63-linked vs. K48-linked polyubiquitination chains. Increased stability of c-Myc enhances the transcription of c-Myc target genes in stem cells, including *Mat2a*, *Smpdl3b*, *Tert*, and *Usp22*. In mESCs, increased expression of MAT2A induces the production of SAM from methionine, which in turn increases H3K4me3 on pluripotent genes and induces their expression. This action is important for the maintenance of pluripotent stem cells. C-Myc in mESCs also induces the expression of SMPDL3B to remodel sphingolipids on the plasma membrane, which impacts membrane fluidity and signaling pathways involved in neuronal differentiation. In post-reprogrammed iPSCs, c-Myc activates the transcription of Tert to promote telomere elongation. In LSCs, c-Myc posttranscriptionally induces overexpression of USP22, a protein deubiquitinating enzyme that can stabilize the SIRT1. This regulation enhances SIRT1-mediated inhibition of p53 while stimulating PGC-1α-mediated mitochondrial biogenesis, promoting LSC survival and proliferation. Figures were created using images downloaded and adapted from Service Medical ART: SMART (https://smart.servier.com/image-set-download/). Servier Medical Art by Servier is licensed under a Creative Commons Attribution 3.0 Unported License (https://creativecommons.org/licenses/by/3.0/).”

One metabolic pathway that is critically involved in epigenetic regulation of stem cell pluripotency is methionine metabolism. As a sulfur-containing essential amino acid, methionine is a key component of dietary proteins important for protein synthesis, sulfur metabolism, epigenetic modification, antioxidant defense, and signaling (Mato et al., 2008). Specifically, SAM, the methyl-donor for histone methyltransferases, is produced from methionine by oligomeric enzyme methionine adenosyltransferase (MAT2) in ESCs (Halim et al., 1999; Shiraki et al., 2014). It has been shown that altered methionine or threonine metabolism induce the fluctuation of intracellular SAM. Such fluctuation influences histone methylation in both mESCs and hESCs, thereby modulating their fate (Shyh-Chang et al., 2013; Shiraki et al., 2014). Through a large scale unbiased metabolomic analysis of SIRT1 KO and control WT mESCs, Tang et al. (2017) discovered that one of primary metabolic defects in SIRT1 deficient mESCs is methionine metabolism, particularly the conversion of methionine to SAM. As a result, SIRT1 deficient mESCs have a reduced cellular SAM abundance and decreased histone methylation levels. Particularly, the levels of H3K4me3, a histone activation mark that is sensitive to methionine deprivation/restriction, is significantly reduced in SIRT1 KO mESCs. This reduction is associated with a dramatic alteration of global gene expression profiles, including reduced expression of a number of pluripotent genes (e.g., *Nanog*). It is also associated with a hypersensitivity to methionine depletion/restriction-induced differentiation and apoptosis. Mechanistically, Tang et al. (2017) showed that SIRT1 promotes SAM production in part through Myc-mediated transcriptional activation of *Mat2a*, which encodes the catalytic subunit of Mat2. Deletion of SIRT1 leads to hyperacetylation of both N- and c-Myc proteins. Hyperacetylation in turn leads to instability of c-Myc and reduced recruitment of both factors to the promoter of *Mat2*, and thereby reducing expression of this enzyme (Figure 3). In support of this notion, adding back MAT2A rescues the reduction of H3K4m3 and *Nanog* mRNA, enhances differentiation, and increases apoptosis upon methionine restriction in SIRT1 KO mESCs. Therefore, the epigenetic homeostasis of mESCs, comprising the methylation status of core histone protein (H3K4me3) and profiles of gene expression, is maintained by the SIRT1-c-Myc axis through regulation of methionine metabolism. Importantly, SIRT1 KO mouse embryos have reduced *Mat2a* expression and histone methylation and are sensitive to maternal methionine restriction-induced lethality. Conversely, maternal methionine supplementation increases the survival of SIRT1 KO newborn mice. All those observations suggest that the defective methionine metabolism is partially responsible for SIRT1 deficiency-induced developmental defects in mice (Tang et al., 2017).

Metabolomic analysis revealed that SIRT1 deficient mESCs also exhibit dramatic accumulation of sphingomyelin independently of the defects in methionine metabolism as previously reported by Tang et al. (2017). Sphingomyelin is a type of sphingolipids, which is a class of natural lipids enriched in central nervous system (Merrill et al., 2007; Chen et al., 2010; Rao et al., 2013). In addition to be main structural components of cell membrane, sphingolipids act as important signaling molecules controlling many cellular events such cell growth, differentiation, and apoptosis (Hannun and Obeid, 2008; van Meer et al., 2008). The significance of sphingolipids for human health is best demonstrated by the observation that many neurodegenerative diseases, such as Niemann-Pick’s, Alzheimer’s, and Parkinson’s, are associated with defects in sphingolipids degradation enzymes and impaired sphingolipid metabolism (Brice and Cowart, 2011; Czubowicz et al., 2019). Particularly, sphingolipids are bioactive lipids critical for survival and differentiation of stem cells (Bieberich, 2008). Fan et al. confirmed that different SIRT1 deficient mESC lines have significantly increased levels of sphingomyelin, primarily due to a marked reduction of sphingomyelin phosphodiesterase acid like 3B (SMPDL3B) (Fan et al., 2021). SMPDL3B is a GPI-anchored plasma membrane bound sphingomyelin phosphodiesterase that degrades sphingomyelin into ceramide. Utilizing ChIP-qPCR assay, promoter analysis, luciferase reporter assay, and sgRNA/dCas9-mediated *in situ* gene expression perturbation, they further found that the *Smpdl3b* promoter is located within a bivalent chromatin domain targeted by c-Myc and EZH2, a H3K27me3 transferase. SIRT1 actively modulates this bivalent domain, primarily through deacetylation and stabilization of c-Myc. Loss of SIRT1 decreases c-Myc binding to the *Smpdl3* promoter, which in turn increases EZH2 recruitment and H3K27me3, resulting in silencing of *Smpdl3b* (Figure 3). Functionally, accumulation of sphingomyelin in SIRT1 KO mESCs disrupts the integrity of cell membrane and subsequently increases the membrane fluidity. The increase of cell membrane fluidity does not significantly impact pluripotency of mESCs, but instead markedly delays and impairs *in vitro* differentiation of mESCs into neural progenitors and mature neurons (Fan et al., 2021). When analyzed *in vivo*, Fan et al. (2021) showed that maternal high-fat diet feeding elevates sphingomyelin contents in all brain regions of SIRT1 KO embryos. This metabolic defect is associated with reduced expression of many markers of intermediate progenitors and mature neurons and delaying intrauterine growth of embryos. This study uncovers a novel function of the SIRT1-c-Myc axis in maintaining sphingolipid homeostasis and normal neural differentiation of mESCs, which are important for normal mouse embryonic development.

Both studies highlight the importance of the SIRT1-c-Myc axis in metabolic and epigenetic regulation of mESC pluripotency, differentiation, and mouse embryogenesis.

## 6 The SIRT1-c-Myc axis promotes telomere elongation of iPSCs

In vertebrates, telomeric repeats (TTAGGG tandem repeats), which constitute a telomere, are synthesized by telomerase expressed mainly in the period of embryonic development and in adult stem cells (de Lange, 2005; Flores et al., 2006a; Liu et al., 2007). During reprogramming of MEFs into iPSCs, telomeres are elongated, and telomere elongation has been recognized as a hallmark of an iPSC (Takahashi and Yamanaka, 2006). The SIRT1-c-Myc axis has been reported to promote telomere elongation of iPSCs (De Bonis et al., 2014).

SIRT1 is extremely highly expressed in mESCs compared with adult stomatic cells and differentiated cells such as MEFs (Calvanese et al., 2010; Tang et al., 2017). De Bonis et al. (2014) showed that during reprogramming from MEFs to iPSCs, the expression of SIRT1 is continuously induced and eventually reaches to a comparable level with that in mESCs. The increased expression of SIRT1 is coupled with the formation of the hyper-long telomeres. Specifically, utilizing loss-of-function (*Sirt1* ^*−/−*^) and gain-of-function (*Sirt1* ^*Super*^) MEFs, they showed that the expression level of SIRT1 does not affect the reprogramming of MEFs. However, telomeres in *Sirt1* ^*−/−*^ iPSCs are significantly shorter than those in *Sirt1* ^*+/+*^ iPSCs, whereas the length of telomeres in *Sirt1* ^*Super*^ is 20% in average longer than that in *Sirt1* ^*−/−*^ iPSCs. Moreover, telomeres in *Sirt1* ^*+/+*^ iPSCs elongate more progressively in the stage of post-reprogramming than those in *Sirt1* ^*−/−*^ iPSCs. Therefore, SIRT1 is required for telomere elongation in the stage of post-reprogramming.

In cancer cells, c-Myc activates the transcription of mouse telomerase reverse transcriptase (*mTert*), the catalytic subunit of telomerase (Wang et al., 1998; Flores et al., 2006b). De Bonis et al. showed that in late-passage iPSCs, SIRT1 increases the stability of c-Myc, which in turn promotes the transcription of *mTert* and telomere elongation (De Bonis et al., 2014). Consequently, SIRT1 deficient iPSCs accumulate chromosomal aberrations and display a derepression of telomeric heterochromatin. Therefore, SIRT1 positively regulates the expression of TERT by enhancing the stability of c-Myc protein (Figure 3).

## 7 The SIRT1-c-Myc axis promotes the maintenance and drug resistance of leukemia stem cells

In acute myeloid patients (AML), self-renewing leukemic stem cells (LSCs) generate a bulk of leukemic cells and correlate with low prognosis (Eppert et al., 2011; Patel et al., 2012). In AML patients containing the internal tandem duplication (ITD) in the Fms-like tyrosine kinase (*FLT3*) gene, lack of elimination of LSCs due to their strong drug resistance is presumably responsible for failed treatment with the small molecules of FLT3 tyrosine kinase inhibitors (TKIs) (Levis, 2011; Horton and Huntly, 2012; Smith et al., 2012).

Li et al. (2014) reported that the positive feedback between SIRT1 and c-Myc contributes to the maintenance and drug resistance of FLT3-ITD AML LSCs. Li et al. (2014) found that SIRT1 is overexpressed in the primary human FLT3-ITD AML LSCs due to c-Myc induced overexpression of USP22, a protein deubiquitinating enzyme that can stabilize SIRT1 (Lin et al., 2012). Increased SIRT1 protein in LSCs in turn inhibits p53 and enhances PGC-1α-mediated mitochondrial biogenesis, promoting LSC survival and proliferation (Li et al., 2014). Conversely, SIRT1 knockdown or inhibition by its inhibitor Tenovin-6 (TV6) increases c-Myc acetylation, enhancing its degradation and subsequent reduction in transcriptional activity in FLT3-ITD cells (Figure 3). In support of the notion that the positive SIRT1-c-Myc feedback loop contributes to partial maintenance of FLT3-ITD AML LSCs after treatment with TKI, inhibition of SIRT1 expression or activities reduces their growth and significantly enhances their sensitivity to TKIs (Li et al., 2014). The findings from this study suggest that targeting the SIRT1-c-Myc axis using the small molecule inhibitors of SIRT1 could potentially improve outcomes of TKI-based treatment of FLT3-ITD AML.

## 8 Concluding remarks

While c-Myc is a well-known oncoprotein, the impact of SIRT1 on tumorigenesis is distinct at different stages depending on its deacetylation substrates, which include both tumor suppressors and oncogenic proteins (Garcia-Peterson and Li, 2021). The positive feedback loop between SIRT1 and c-Myc has been reported to suppress senescence and apoptosis in established cancer cells (Menssen et al., 2012). Recent studies revealed that this positive feedback loop is particularly important in maintenance, proliferation, and stress resistance of stem cells, including PSCs and CSCs. In PSCs, these actions are crucial for the maintenance of their pluripotency, self-renewal, and differentiation, which are ultimately important for normal embryogenesis. In CSCs, the impacts of SIRT1-c-Myc axis could result in drug resistance, relapse, and metastasis of tumors, thereby directly influencing therapeutic outcomes. Future studies are still needed to better understand the functional importance of the SIRT1-c-Myc axis in different type of stem cells. In particular, the maintenance and early lineage specification of primed hESCs are regulated by signaling pathways such as FGF and Activin/Nodal signaling (Brown et al., 2011; Fathi et al., 2017). Yet the potential role of the SIRT1-c-Myc axis in regulation of these signaling in hESCs remains unknown. Future research along this line could provide molecular basis for novel therapeutic strategies against developmental diseases and cancers.

## Abbreviation

DBC1, deleted in breast cancer 1; Dnmt3l, DNA methyltransferase 3-like; FLT3, Fms-likes tyrosine kinase; iPSCs, induced pluripotent stem cells; ITD, internal tandem duplication; LIF, leukemia inhibitory factor; LSC, leukemic stem cells; MAL, acute myeloid leukemia; MAT2, methionine adenosyltransferase; MAT, methionine adenosyltransferase; MEFs, murine embryonic fibroblasts; mESCs, murine embryonic stem cells; NAD, nicotinamide adenosine dinucleotide; NAMPT, nicotinamide-phosphoribosyltransferase; NSCs, neural stem cells; PSCs, pluripotent stem cells; SAM, S-adenosylmethionine; SM, sphingomyelin; SMPDL3B, sphingomyelin phosphodiesterase acid like 3B; STAT3, Signal transducer and activator of transcription 3; Tert, Telomerase reverse transcriptase; TSS, Transcription start site.
